# Sex-Specific Social Behavior and Amygdala Proteomic Deficits in *Foxp2*^+/−^ Mutant Mice

**DOI:** 10.3389/fnbeh.2021.706079

**Published:** 2021-08-05

**Authors:** Maria Jesus Herrero, Li Wang, David Hernandez-Pineda, Payal Banerjee, Heidi Y. Matos, Meredith Goodrich, Aswini Panigrahi, Nathan Anthony Smith, Joshua G. Corbin

**Affiliations:** ^1^Center for Neuroscience Research, Children’s National Hospital, Washington, DC, United States; ^2^Center for Genomic Medicine, Children’s National Hospital, Washington, DC, United States; ^3^Center for Cancer and Immunology Research, Children’s National Hospital, Washington, DC, United States

**Keywords:** *Foxp2*, medial amygdala (MeA), social behavior, aggression, sex-specific differences, proteomics

## Abstract

In humans, mutations in the transcription factor encoding gene, *FOXP2*, are associated with language and Autism Spectrum Disorders (ASD), the latter characterized by deficits in social interactions. However, little is known regarding the function of *Foxp2* in male or female social behavior. Our previous studies in mice revealed high expression of Foxp2 within the medial subnucleus of the amygdala (MeA), a limbic brain region highly implicated in innate social behaviors such as mating, aggression, and parental care. Here, using a comprehensive panel of behavioral tests in male and female *Foxp2*^+/–^ heterozygous mice, we investigated the role *Foxp2* plays in MeA-linked innate social behaviors. We reveal significant deficits in olfactory processing, social interaction, mating, aggressive, and parental behaviors. Interestingly, some of these deficits are displayed in a sex-specific manner. To examine the consequences of *Foxp2* loss of function specifically in the MeA, we conducted a proteomic analysis of microdissected MeA tissue. This analyses revealed putative sex differences expression of a host of proteins implicated in neuronal communication, connectivity, and dopamine signaling. Consistent with this, we discovered that MeA *Foxp2*-lineage cells were responsive to dopamine with differences between males and females. Thus, our findings reveal a central and sex-specific role for *Foxp2* in social behavior and MeA function.

## Introduction

In humans, the function of the transcription factor encoding gene, *FOXP2*, was first primarily associated with speech, language production, and vocal communication. Several different mutations in *FOXP2* result in difficulty in coordinating speech-related movements (developmental verbal dyspraxia, dysarthria, and/or apraxia of speech), as well as deficits in receptive and expressive verbal abilities (dysphasia), first described in the KE family (Vargha-Khadem et al., 1998; Lai et al., 2001; MacDermot et al., 2005; Feuk et al., 2006; Shriberg et al., 2006; Zeesman et al., 2006; Fisher and Scharff, 2009; Morgan et al., 2017). Based on these findings, *FOXP2* was initially dubbed “the language gene.” These findings prompted a myriad of investigations in mouse models into the function of *Foxp2*, as well as in other species including songbirds, bats, and non-human primates. The majority of these studies focused on sensory and motor-related brain regions implicated in language/verbal communication such as the basal ganglia (caudate nucleus and striatum), cerebellum, and the cerebral cortex, all regions with high expression of *Foxp2* (Haesler et al., 2004; Shu et al., 2005; Vargha-Khadem et al., 2005; Li et al., 2007; Fujita et al., 2008; Groszer et al., 2008; Enard et al., 2009; Konopka et al., 2009, 2012; Gaub et al., 2010; Hilliard et al., 2012; Castellucci et al., 2016).

In addition to these studies, another line of investigation has unveiled interesting correlations between *FOXP2* mutations and social impairment. Large chromosome mutations comprising the *FOXP2* region or polymorphisms in the* FOXP2* gene have been associated with social deficits and autistic phenotypes; some of them concomitant with language deficits (Gong et al., 2004; Li et al., 2005; Marui et al., 2005; Feuk et al., 2006; Laroche et al., 2008; Konopka, 2013; Medvedeva, 2015; Chien et al., 2017; Morgan et al., 2017; and see DECIPHER and SFARI databases). The exact role of *FOXP2* in Autism Spectrum Disorders (ASD) remains to be fully determined, as several rounds of large scale genome-wide sequencing studies showed no association with ASD (Newbury et al., 2002; Gauthier et al., 2003; Kumar, 2008; Marshall et al., 2008; Weiss et al., 2008; Wang et al., 2009; Abrahams and Geschwind, 2010; Anney et al., 2010; Krishnan et al., 2016). However, more recent studies, most notably the large-scale sequencing effort by Satterstrom et al. (2020) have uncovered a correlation between *FOXP2* mutations and ASD (Lim et al., 2017; Guo et al., 2019; Munnich et al., 2019; Satterstrom et al., 2020). Although more and larger sequencing studies will be informative, based on the current evidence, the Simons Foundation Autism Research Initiative (SFARI GENE) ranks *FOXP2* as a category 1, “high confidence” ASD gene (following the highest confidence category of “Syndromic”). Consistent with the role of *Foxp2* in social behavior, studies in mouse models also reported diminished social function in several *Foxp2* mutants. For instance, heterozygous *Foxp2*^+/–^ mice were less frequently in group contact in the modified Hole Board test (Shu et al., 2005; Enard et al., 2009). Also, cortical-specific *Foxp2* conditional mutant mice showed deficits in approach towards conspecifics (Medvedeva, 2015; Medvedeva et al., 2019).

Despite the above described extensive studies of *Foxp2* in different brain areas mainly involved in language, studies of *Foxp2* function in the amygdala, a central brain region implicated in social behavior and communication, have been lacking. In the amygdala, Foxp2 is highly expressed in both the intercalated neurons (ITCs), which are associated with fear conditioning, and the medial subnucleus (MeA), which is essential for processing olfactory cues that trigger innate (unlearned) behaviors such as mating, aggression, parenting, and predator avoidance (Lischinsky et al., 2017; Kuerbitz et al., 2018; Lischinsky and Lin, 2020). Our previous studies using either immunohistochemistry to label Foxp2+ cells or Cre-based fate mapping to tag Foxp2+ cells and their descendants revealed that Foxp2+ and *Foxp2*-lineage neurons in the MeA are a molecular and electrophysiologically distinct subpopulation of inhibitory output neurons (Carney et al., 2010; Lischinsky et al., 2017; Matos et al., 2020). Our work further revealed that Foxp2+ and *Foxp2*-lineage neurons are activated by innate reproductive and aggressive behaviors in a sex-specific manner and display molecular and intrinsic electrophysiological differences between males and females (Lischinsky et al., 2017; Matos et al., 2020).

In addition to our findings, other studies have revealed sex differences in *Foxp2* function in different brain regions, demonstrating that at least some of the roles of *Foxp2* are sex-specific. For instance, in rats, the level of *Foxp2* expression correlates with male and female differences in ultrasonic vocal communication by pups, leading to a dam’s preferential retrieval of male pups (Bowers et al., 2013). In addition, the male sex hormone androgen promotes Foxp2 expression in the striatum, cerebellar vermis, and cortex (Bowers et al., 2014).

Based on these findings, we explored the putative role *Foxp2* plays in the modulation of MeA driven innate social and non-social behaviors in males and females by examining *Foxp2*^+/–^ mice. We found sex differences in a subset of innate behaviors, including most strikingly opposite effects on aggressive behaviors in males and females. Correlating with these sex differences in behavioral alterations, we found putative male/female differences in alterations at the protein level in *Foxp2*^+/–^ mice in the MeA. These include changes in proteins implicated in DA-signalling, which has previously been associated with MeA-driven motivational behaviors (Miller et al., 2019; Nordman and Li, 2020). We further found that MeA *Foxp2*-lineage cells are highly responsive to dopamine, also in a sex-specific manner. Thus, our study uncovers a previously unknown role for *Foxp2* in regulating innate behaviors and potentially in motivational states in male and female mice; processes perhaps contributed *via* *Foxp2* function in the MeA.

## Materials and Methods

### Animals and Housing

Mice were housed in the temperature and light-controlled (12 h light-dark cycle) animal care facility at Children’s National Hospital and given food and water *ad libitum*. All animal procedures were approved by the Children’s National Hospital Institutional Animal Care and Use Committee (IACUC) and conformed to NIH Guidelines for animal use. *Foxp2*^+/–^ mutant mice used in this study were previously generated by replacing exons 12 and 13 with a neomycin cassette resulting in a complete loss of function in homozygous *Foxp2*^−/−^ mice (Shu et al., 2005). As *Foxp2*^−/−^ mice are early postnatal lethal, only *Foxp2*^+/–^ mice were used here. *Foxp2*^+/–^ mice were maintained on the C57BL/6J background. *Foxp2*^+/–^ mice were generated from crossing to *Foxp2*^+/–^ males to C57BL/6J females. *Foxp2*^+/–^ mice are termed “mutant” (mt) with controls (ctr) wild-type littermates or C57BL/6J mice. *Foxp2^Cre^* mice (JAX labs Stock No: 030541; Rousso et al., 2016) and *GCaMP5G-tdTm* mice (Jax Labs Stock No: 024477; Gee et al., 2014) were obtained from Jackson Labs. Mice were genotyped by Transnetyx Inc. Genotyping Services. Mouse chow was standard diet (Teklad global rodent diets, #2918, 18% protein, Envigo, Indianapolis, IN, USA) and bedding was Corncob 1/4, #7097 (Envigo, Indianapolis, IN, USA).

### Behavioral Experiments

Behavioral experiments were conducted using two to three separate cohorts of 3–4 month-old male and female *Foxp2*^+/–^ mutants and control mice with a total *n* ≥ 10 animals per group (exact *n*’s noted in the *Results and/or Figure Legends*). For behavioral analyses, each cohort roughly included 3–5 pups/sex/genotype/litter from approximately 5 to 6 litters. Video tracking was used in the analysis of social behavior (Dold Labs and Engineering software) and the other behaviors tracked by visual observation and scoring behavioral data by an observer blind to the genotype of the animals. Experiments were carried out in the Children’s National DC-IDDRC (1U54HD090257) supported Animal Neurobehavioral Evaluation Core (ANEC).

### Social Interaction Assay

Social behavior was evaluated using an automated 3-chamber device (Dold Labs and Engineering, Seguin, TX, USA), as previously described (McFarlane et al., 2008). The socialization chamber is composed of three partitions separated by retractable doorways. On test days, a single *Foxp2*^+/–^ or ctr mouse was placed in the middle chamber and allowed to explore freely for 10 min. After 10 min, the mouse was given free access to the other two chambers for 10 min. After this exploratory period, an inverted empty wire cup (novel object) was placed in one of the chambers, and an unfamiliar (novel) C57BL/6J sex- and age-matched mouse (i.e., males with males and females with females) was placed inside an identical inverted wire cup in the other chamber. In order to control for the possibility of innate side preferences, the wire cups with and without the novel mouse were randomly placed on the left or right chambers, and the placement was changed between mice (Wang et al., 2015). Animal behaviors were video-recorded for 10 min and the total time the experimental mouse spent sniffing the novel mouse, the novel object, and the total time spent in each chamber were scored.

### Olfactory Habituation and Discrimination Assay

Urine pooled from multiple female mice across the estrus cycle, pooled male urine, peanut butter, or water were swabbed onto a cotton swab. Each odor-swabbed cotton swab was presented to *Foxp2*^+/–^ or ctr mice three times each for 2 min, with 2 min between each inter-trial interval. Active sniffing was scored each time the animal’s nose was oriented towards and less than 2 cm from the tip of the swab. The cumulative time spent investigating a particular odor during the 2 min trial was scored.

### Mating Assay

Sexually naïve experimental *Foxp2*^+/–^ and control males and females were singly housed for 1 week prior to testing. A sexually experienced C57BL/6J female or male, respectively, was placed into the test male or female home-cage. Each test trial was recorded for 30 min. Trials were repeated three times, separated by at least 1 week (total trials: 60 for males and 66 trials for females). The number, duration, and latency of the mating parameters of mounting, intromission, and ejaculation were scored using criteria previously described by us (Sokolowski et al., 2015).

### Maternal Aggression Assay

Singly housed *Foxp2*^+/–^ or control female mice were impregnated *via* cross to a C57BL/6J male and gave birth to a litter of pups. Tests were conducted when pups were between P3-P10. On the test day, pups were removed from the home-cage, and an intruder C57BL/6J male was introduced. Each trial consisted of 15 min of interaction. The time and number of nips (pinching with the mouth) and attacks were recorded and scored. Nips were defined as pinching with the mouth, without other motor components. Attacks were defined as the full repertoire of the motor components of aggression such as biting combined with clinching and tussling. The pups were returned to the home-cage after the intruder was removed. Trials were repeated two times, separated by at least 1 week.

### Resident-Intruder Aggression Assay

Each resident *Foxp2*^+/–^ or control male was singly housed and undisturbed for at least 1 week prior to testing. After this time, a smaller novel C57BL/6J male was introduced into the home-cage of the resident. Each trial was recorded for 10 min. The time and number of social explorations (moving directly toward conspecific, chasing, or sniffing) and attacks (fighting and clinching in an upright position) were scored.

### Pup Retrieval Test

*Foxp2*^+/–^ or control mice gave birth to a litter of pups. In each trial, the dam was temporarily removed from the cage, and the litter of P3-P10 pups was taken from the nest and placed in a far corner of the home-cage. The dam was returned to the cage, and the time required for her to retrieve the first and second pup back to the nest was scored. Trials were repeated two times, separated by at least 1 week.

### Predator Avoidance Assay

The predator odor consisted of a plastic petri dish containing rat bedding (12g), introduced into the home-cage. The benign odor consisted of a petri dish containing clean unsoiled mouse bedding (12g). To habituate the mice to the dish, a novel object, *Foxp2*^+/–^ and control mice were exposed to benign odor for 15 min. Then, the predator odor rat bedding in a petri dish was presented into the home-cage for 15 min. The escape behaviors (climbs) and chemoinvestigative behaviors (risk assessments, as defined by cautious investigation, and sniffs) were scored.

## Proteomics: Relative Protein Quantitation Using TMT Isobaric Mass Tagging

Adult 3-month-old male and female *Foxp2*^+/–^ and control mice were euthanized, and the MeA encompassing both the MePD and MePV (Bregma −1.58 to −2.06; Mouse Brain Atlas, 3rd edition; Franklin and Paxinos, 2008) was microdissected manually using Lumdsen microdissection scissors. The MeA is observed as a teardrop structure distinct from nearby amygdala nuclei. Care was taken to avoid dissecting nearby amygdala nuclei (CeA, CoA), notably avoiding Foxp2+ intercalated neurons that are found adjacent to the basolateral amygdala which is bounded by clearly demarcating white matter tracts. Dissected tissue was snap-frozen in liquid nitrogen and stored at −80°C until processed. MeA tissue from three animals (two amygdala/animal for a total of six) was pooled together and considered a single sample. A total of *n* = 2 samples were analyzed for each sex (male and female) and genotype (*Foxp2*^+/–^ and control). MeA tissue samples were homogenized in 8 M urea and sonicated for 30 s. The resultant lysate was centrifuged at 16K rpm for 30 min at 4°C and cleared supernatant was collected. Protein concentration was determined by BCA assay (Pierce^TM^, Thermo Scientific), and 100 μg of protein lysate was processed for each sample. The proteins were treated with TCEP and Iodoacetamide as described in TMT Isobaric Mass Tags labeling protocol (Thermo Scientific), extracted with methanol: chloroform, air dried, dissolved in 50 mM TEAB, and digested overnight with sequencing grade Trypsin (Thermo Scientific) at 37°C. The resulting peptides were labeled with TMT Label Reagent (Sixplex kit, Thermo Scientific), each with one specific tag (as per manufacturer’s protocol). Following labeling, the samples were pooled together, desalted, and fractionated to eight fractions using Pierce high-pH fractionation kit (Thermo Scientific). The peptide mixtures from each fraction were sequentially analyzed by LC-MS/MS using nano-LC system (Easy nLC1000) connected to Q Exactive HF mass spectrometer (Thermo Scientific). The platform is configured with nano-electrospray ion source (Easy-Spray, Thermo Scientific), Acclaim PepMap 100 C18 nanoViper trap column (3 μm particle size, 75 μm ID x 20 mm length), EASY-Spray C18 analytical column (2 μm particle size, 75 μm ID x 500 mm length). The peptides were eluted at a flow rate of 300 nl/min using linear gradients of 7–25% Acetonitrile (in aqueous phase and 0.1% Formic Acid) for 80 min, followed by 45% for 25 min, and static flow at 90% for 15 min. The mass spectrometry data was collected in a data-dependent manner switching between one full scan MS mode (m/z 380–1,400, resolution 60K, AGC 3e^6^, max ion time 20 ms), and 10 MS/MS scans (resolution 15K, AGC 1e^5^, max ion time 120 ms, nCE 32) of the top 10 target ions. The ions were sequenced once and dynamically excluded from the list for 30 s. The MS raw data sets were analyzed using Thermo Proteome Discoverer Software (version 2.3), and searched against the Mouse UniProt database using Sequest HT algorithm at precursor mass tolerance of 10 ppm and fragment mass tolerance of 0.02 Da. The peptides and proteins were filtered using a Percolator at a target FDR of 0.01 (thus at 99% confidence level). The relative quantitation of proteins was determined based on the intensities of reporter mass tags. We used open-source Perseus software for data filtration to exclude any false hits and to select only high confidence quantified proteins. Heatmap visualization of the dataset based on relative protein abundance ratio between the two groups of animals was done by Hierarchical clustering analyses using Euclidean distance, with maximum of 10 iterations. As we are using three animals in each group, the abundance represents the average of three animals. Proteins with similar abundance between the two independent groups of analyses were selected for downstream analyses. We note that in this study we performed global quantitative proteomics analyses only in two technical replicates, thus the results from this experiment should be viewed as a hypothesis-generating analysis rather than one that conclusively validates the differences in specific proteins.

## Dopamine Activation of MeA *Foxp2*-Lineage Cells *Ex vivo*

### Acute Brain Slice Preparation

Three- to 7-month-old male and female *Foxp2^cre^;GCaMP5G-tdTM* mice were euthanized, and brains rapidly removed and immersed in ice-cold cutting solution (230 mM sucrose, 2.5 mM KCl, 0.5 mM CaCl_2_, 10 mM MgCl_2_, 26 mM NaHCO_3_, 1.25 mM NaH_2_PO_4_, 0.04 mM Na-Ascorbate, and 10 mM glucose, pH 7.2–7.4). Coronal slices containing the MeA (Bregma −1.58 to −2.06) were cut with a vibratome (Leica VT1000S) at 300 μm and transferred to artificial cerebral spinal fluid (aCSF; 126 mM NaCl, 4 mM KCl, 2 mM CaCl_2_, 2 mM MgCl_2_, 26 mM NaHCO_3_, 1.25 mM NaH_2_PO_4_, 0.04 mM Na-Ascorbate, and 10 mM glucose, pH 7.2–7.4, osmolarity = 310 mOsm/l) bubbled with 95% O_2_ and 5% CO_2_. Slices were allowed to recover in oxygenated aCSF for 1 h at room temperature before acute slice imaging. During imaging, slices were placed in a perfusion chamber and superfused with oxygenated aCSF at 30° to 32°C for the duration of the experiment. The cells were visualized with a 20× immersion objective (Olympus Optical, New York, NY) and epifluorescence.

### Ca^2+^ Imaging and Analysis

Two-Photon Ca^2+^ imaging was performed with an Olympus (Tokyo, Japan) FluoView FVMPE-RS Multiphoton Microscope imaging system using FluoView software and a Ti: Sapphire laser source emitting 140 fs pulses at an 80 MHz repetition rate with a wavelength adjustable for 690–1,040 nm (Maitai DeepSee pulsed, infrared laser). The full field of view was acquired with XY raster scanning using the 20× 0.95 NA water-immersion objective. Changes in fluorescence (ΔF) were quantified using ImageJ (NIH) software and expressed as a percentage of baseline (%ΔF/F). Time-lapse images of cellular Ca^2+^ signaling were recorded at a frame rate of 1 Hz. ROIs were selected based on the appearance of *GCaMP5G* Ca^2+^ transients in the time-lapse images. To trigger Ca^2+^ transients, dopamine (100 μM) was dissolved in aCSF and delivered locally by a pressure pulse (10 psi; 100–500 ms) using a Picospritzer III (Parker Instrumentation, Chicago, IL).

## Statistical Analyses

### Behavioral Experiments

Statistical analyses and graph plots were carried out using Microsoft Excel and GraphPad (Prism9). Data were represented by the mean ± S.E.M. (standard error of the mean). Significant differences were revealed by either unpaired *t*-test for 2-group mean comparisons or by ANOVA for multiple comparisons using Tukey’s *post hoc* test, after verifying normality and homoscedasticity by Shapiro–Wilk’s and Levene’s test, respectively.

### Proteomics

Proteomics analysis was carried out in two samples per condition, i.e., per male and female *Foxp2*^+/–^ mutants (mt) and controls (wild type siblings). Each sample represented pooled MeA from *n* = 3 animals. For analyzing differential protein expression in *Foxp2*^+/–^ mutant vs. wild type siblings (ctr) in males and females, relative expression was assessed by generating the ratios: mtM1/ctrM1, mtM1/ctrM1 and mtF/mtM. Log2FC ≥ |1.11| change was considered significant. Proteins were clustered based on similarity of expression between the experimental and control groups, which is indicated by the dendrogram. The color key and histogram panel indicates the *Z*-scores of expression and the expression counts. The related pathways, biological processes, and ontology of the differentially expressed proteins were determined using Ingenuity Pathway Analysis software (IPA, Qiagen vs. 2020) and the Cytoscape (vs. 3.5.1) plug-in ClueGO (vs. 2.5.5). Heatmaps were generated using R (version 3.6.2) with the heatmap.2 function in the gplots package (version 3.1.1). Map colors used the RColorBrewer palette (version 1.1-2). Proteins were clustered based on similarity of expression between the experimental and control groups, indicated by the dendrogram. Highly expressed proteins are shown in red, while those with lower expression are shown in green. Differentially expressed proteins in the MeA of *Foxp2*^+/–^ mutants were then compared with categories “Syndromic”, “1. High confidence”, “2. Strong candidate” and “3. Suggestive evidence” of the publicly available SFARI list reporting autism-susceptibility genes (version release 04-13-2020, by the Simons Foundation Autism Research Initiative), as well as with the largest exome sequencing study of ASD to date (Satterstrom et al., 2020). To visualize overlap between comparisons, Venn diagrams were created using an online tool (Oliveros, 2007).

### Dopamine Activation

Activation of *Foxp2*-lineage cells by dopamine was investigated in at least two MeA slices from five *Foxp2^cre^;GCaMP5G- tdTM* per sex. All values are expressed as means ± S.E.M. Normality of the data was evaluated with Shapiro–Wilk test with p = 0.05. Differences between the two means were assessed by Student’s test (unpaired; Mann–Whitney *post hoc* test). Statistical significances (p* ≤ 0.05) were calculated using GraphPad Prism 6.0 (GraphPad Software, La Jolla, CA, USA).

## Results

### Alterations in Social and Non-social Innate Behaviors in *Foxp2*^+/–^ Mice

Previous studies revealed that *Foxp2* is expressed in areas of the brain, such as the amygdala, that regulate social behavior (Co et al., 2020). Therefore, we wanted to examine whether *Foxp2*^+/–^ mutant mice display alterations in amygdala regulated social and non-social innate behaviors and if so, whether there are differences across males and females. To test this, we conducted the following tasks: the 3-chamber social test, olfactory habituation/dishabituation, mating/reproduction, female maternal and male territorial resident-intruder aggression, pup retrieval, and predator odor avoidance. As described below, we found robust deficits in a number of behavioral paradigms tested, a subset which also differed between males and females.

#### Three-Chamber Social Test

**Figure 1:**
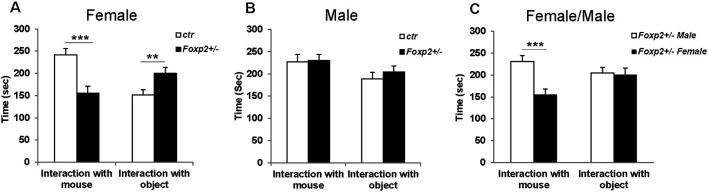
Decreased social interaction in female *Foxp2*^+/–^ mice in the 3-chambered task. Compared to control mice, female *Foxp2*^+/–^ mice show a significant decrease in time spent in the presence of a social cue (mouse; *p* = 0.0004, *Foxp2*^+/–^ *n* = 13, ctr *n* = 12), with a correspondingly significant increase in time spent in the presence of a non-social object (wire cup; *p* = 0.01) **(A)**. In contrast, male *Foxp2*^+/–^ mice showed no significant changes in time spent in social or non-social interactions compared to control (*p* = 0.89, *Foxp2*^+/–^ *n* = 13, ctr *n* = 12) **(B)**. When directly comparing male and female *Foxp2*^+/–^ mice, a significant decrease was observed in social interaction in females compared to males, with no change in non-social interaction (**C**; p** ≤ 0.01; p*** ≤ 0.001).

First, to determine whether *Foxp2* is required for social discrimination, we used the 3-chambered social task, which is considered the gold standard behavioral assay to assess sociability (McFarlane et al., 2008; Wang et al., 2015; Kazdoba et al., 2016). We compared the preference of the test mouse (*Foxp2*^+/–^ or C57BL/6J control) to a social object (male or female mouse) vs. a non-social object (wire cup). In this assay, control mice typically show a greater preference for engaging a novel mouse as opposed to a novel object. The test mouse was placed in the center chamber and allowed to explore freely. Placed in the other chambers were either a wire cup without a mouse and a wire cup with the same sex mouse. Our results showed that female *Foxp2*^+/–^ mice spent less time in the presence of another mouse from the same sex, and instead spent more time in the presence of the object, indicative of deficits in sociability (Figure 1). In contrast, we observed no differences in sociability in male mice. Thus, in the 3-chamber tests, social behavior in *Foxp2*^+/–^ mice was impaired in females, but not in males.

#### Olfactory Habituation/Dishabituation

**Figure 2:**
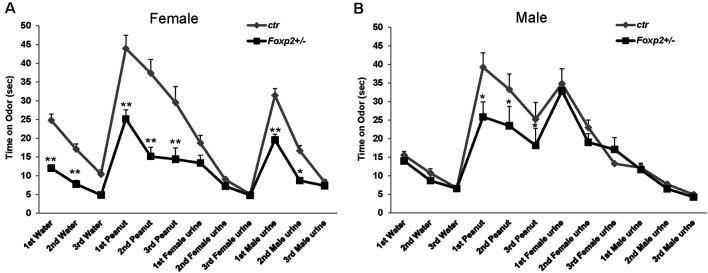
Female and male *Foxp2*^+/–^ mice display decreased time spent investigating different odors. Mice were exposed to different odors (e.g. neutral: water; non-social: peanut butter; and social: female or male urine) three times each for 2 min (with intervals of 2 min between odor exposure). Both female and male *Foxp2*^+/–^ mice spent significantly less time sniffing a non-social motivating odor (peanut) than control mice **(A,B)**. Additionally, female *Foxp2*^+/–^ mice spent significantly less time sniffing both a neutral odor and a social motivating odor (male urine) **(B)**. The experiment was repeated in two different cohorts (*Foxp2*^+/–^ *n* = 13, ctr *n* = 12; p* ≤ 0.05; p** ≤ 0.01).

Using the well-established habituation/dishabituation test (Yang and Crawley, 2009), we next assessed the ability of *Foxp2*^+/–^ mice to detect and discriminate between non-social and social odors. In this task, a mouse is presented with a series of neutral odor (water) followed by attractive odors (peanut butter, urine) to which the animal typically spends more time investigating *via* sniffing. It is expected that mice will habituate to a previously displayed cue with a decrease in sniffing upon repeated presentation of the same cue, and dishabituate with an increase in sniffing when a novel odor is presented. In this assay, both female and male *Foxp2*^+/–^ mice displayed decreased time sniffing olfactory cues; however, this deficit appeared more pronounced in females (Figure 2). Female *Foxp2*^+/–^ mice spent less time than control littermates sniffing both non-social and social motivating odors, and interestingly also water, the neutral odor. However, while female *Foxp2*^+/–^ mice displayed less interaction time with a given cue, habituation/dishabituation appeared to follow the same pattern as control. In contrast, male *Foxp2*^+/–^ mice displayed a significant defect only in response to peanut butter, an appetitive cue, with no differences in time spent sniffing male or female urine. Thus, while habituation/dishabituation still occurred, both female and male *Foxp2*^+/–^ mice appear to have complex deficits in olfactory processing that may relate to deficits in motivation to investigate novel cues and/or a diminished ability to process specific olfactory cues.

#### Mating Assays

**Figure 3:**
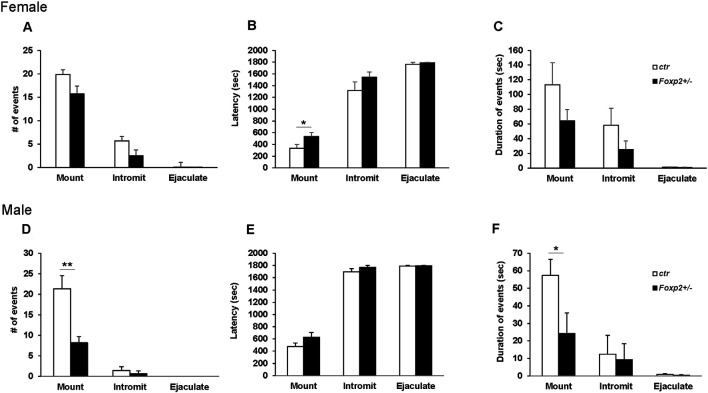
Impaired mating behavior in *Foxp2*^+/–^ mice. The mating repertoire of mounting, intromission, and ejaculation was recorded over a 30 min period. While the number of mounts was unchanged in female *Foxp2*^+/–^ mice, male *Foxp2*^+/–^ mice displayed a decreased number of mounts compared to controls **(A–D)**. In contrast, female *Foxp2*^+/–^ mice displayed a greater latency to mount than controls, a phenotype that was not observed in male *Foxp2*^+/–^ mice **(B,E)**. The duration of events was unchanged in female *Foxp2*^+/–^ mice **(C)**, with a decrease in the duration of mounts in male *Foxp2*^+/–^ mice compared **(F)**. All other parameters were unchanged **(A–F)**. Experiment was repeated in two different cohorts (total females: *Foxp2*^+/–^ *n* = 14, ctr *n* = 13; total males: *Foxp2*^+/–^ *n* = 10, ctr *n* = 10). Trials were repeated three times separated by at least 1 week (total of 81 trials for females, 60 trials for males). p* ≤ 0.05; p** ≤ 0.01.

We next wanted to assess the ability of *Foxp2*^+/–^ mice to execute the mating repertoire. In mice, mating behavior is characterized by stereotypical steps of male chemoinvestigation, followed by mounting, intromission and ejaculation. While both female and male *Foxp2*^+/–^ mice were able to mate, each displayed deficits in specific aspects of the mating repertoire, with females and males displaying different deficits (Figure 3). Specifically, female *Foxp2*^+/–^ mice displayed deficits in latency to mount. In contrast, the number and duration of mounts were significantly lower in *Foxp2*^+/–^ males.

#### Female and Male Aggression

**Figure 4:**
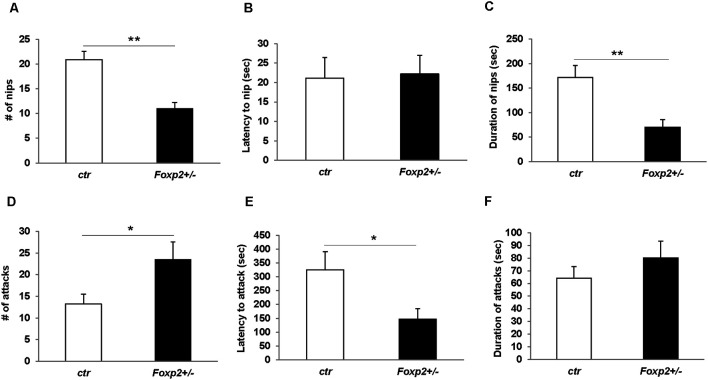
Increased maternal aggression in *Foxp2*^+/–^ mice. An intruder C57BL/6J male was introduced for 15 min to the home-cage of either a lactating *Foxp2*^+/–^ or ctr female mice after removal of pups. Female* Foxp2*^+/–^ mice displayed a decreased number and duration of nips **(A,C)**, but no change in the latency of nips compared to control **(B)**. In contrast, the number and latency of attacks was significantly increased **(D,E)** with no change in the duration of attacks **(F)**. Trials were repeated two times separated by at least 1 week (*Foxp2*^+/–^ *n* = 10, ctr *n* = 12). p* ≤ 0.05; p** ≤ 0.01.

**Figure 5:**
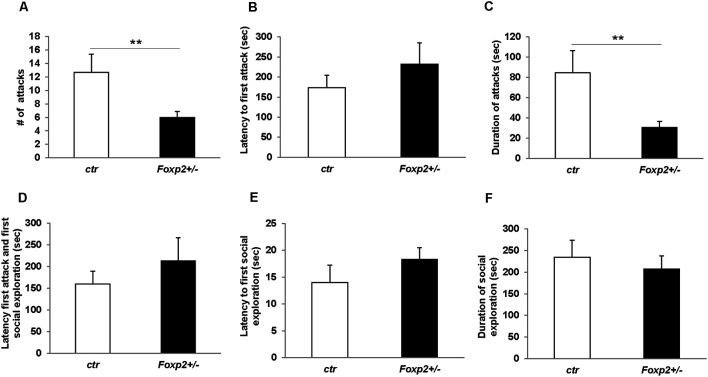
Decreased male territorial aggression in *Foxp2*^+/–^ mice. An intruder C57BL/6J male was introduced into the home-cage of either a *Foxp2*^+/–^ or ctr male for 10 min. Male *Foxp2*^+/–^ mice displayed a significant decrease in both the number and duration of attacks compared to ctr **(A,C)**. However, male* Foxp2*^+/–^ mice did not display any differences in latency to first attack or social exploration, or difference in latency to first attack or social exploration compared to ctr **(B,D,E)**. The total duration of social exploration was also unchanged **(F)** (*Foxp2*^+/–^ *n* = 10, ctr *n* = 10). p** ≤ 0.01.

We next explored maternal aggression and resident-intruder aggression in which female and male mice display aggressive behaviors, respectively (Silverman et al., 2010). Maternal aggression was assessed by placing an intruder mouse in the cage of a dam with pups removed (Figure 4). In this assay, we observed that *Foxp2*^+/–^ dams were more aggressive showing less latency to first attack and attacking more times than the C57BL/6J controls. This was combined with fewer nips, a less aggressive display than a full attack. Thus, female *Foxp2*^+/–^ mice were more overly aggressive to an intruder than controls. In contrast, male *Foxp2*^+/–^ mice showed significantly less aggression when compared to controls, with a decrease in the number and duration of the attacks compared to controls (Figure 5). Interestingly, this decreased aggression was not due to differences in latency to first attack or changes in social exploration, as male *Foxp2*^+/–^ mice investigated the intruder in a similar manner as controls. Thus, female and male *Foxp2*^+/–^ mice display strikingly opposite alterations in aggressive behavior.

#### Pup Retrieval and Maternal Care

**Figure 6:**
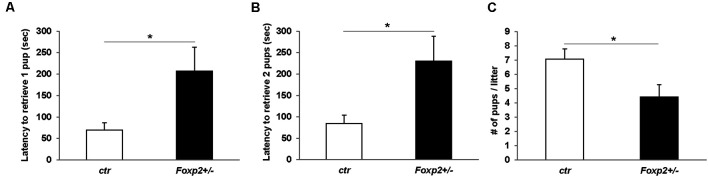
Female *Foxp2*^+/–^ mice display deficits in maternal behavior. To examine pup retrieval, a *Foxp2*^+/–^ or ctr dam was removed from the cage and their pups were removed from the nest and placed in a far corner. When placed back into the home-cage, *Foxp2*^+/–^ dams displayed a significantly greater latency to retrieve one or two pups than ctr dams **(A,B)**. Additionally, female *Foxp2*^+/–^ mice had significantly less pups per litter **(C)**. For **(A,B)**. Trials were repeated two times separated by at least 1 week in two different cohorts (total number of dams: *Foxp2*^+/–^ *n* = 10, ctr *n* = 13; one litter per dam). p* ≤ 0.05.

The ability of a dam to retrieve pups back to her nest is a well-characterized assay for maternal care (Silverman et al., 2010). Using this assay we tested *Foxp2*^+/–^ females’ maternal behavior. We observed that the time to retrieve either one or two pups and bring them back to the nest was significantly greater in *Foxp2*^+/–^ females (Figure 6). This indicates a deficit in motivation to retrieve pups and/or deficits in perceiving or processing sensory input related to pup calls. We further found a decrease in the number of pups per litter from a *Foxp2*^+/–^ dam (Figure 6). We also anecdotally observed more pups found dead in the cage of *Foxp2*^+/–^ dams than with control mice. Together, these results suggest diminished maternal care in *Foxp2*^+/–^ females.

#### Predator Odor Avoidance

**Figure 7:**
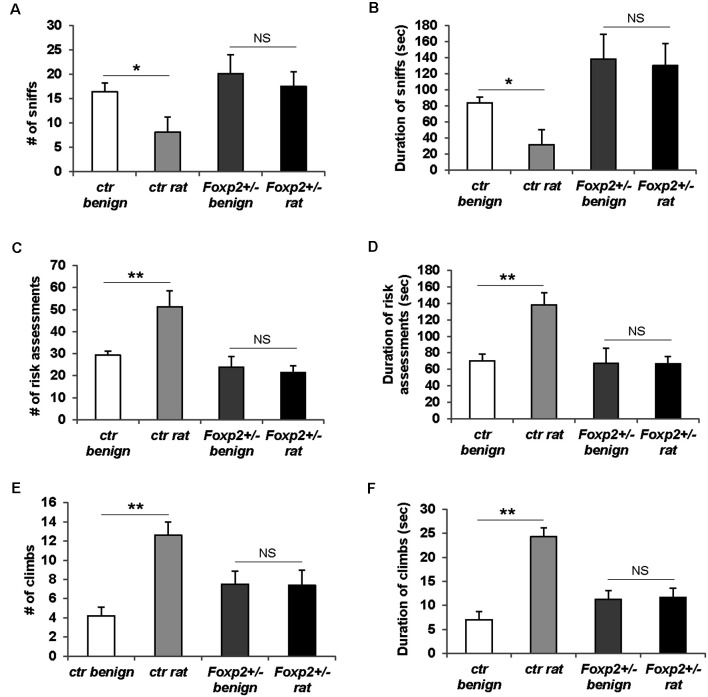
Females *Foxp2*^+/–^ mice display impaired predator odor avoidance. Ctr female mice exposed to the aversive odor of rat bedding display significantly decreased number **(A)** and duration **(B)** of sniffs to the aversive odor when compared to a benign odor (unsoiled bedding). In contrast, female *Foxp2*^+/–^ mice do not display a change in either number **(A)** or duration **(B)** of sniffs to rat bedding compared to a benign odor. Ctr female mice also display the stereotypical risk assessment behavior to rat bedding, as defined by an increased number **(C)** and duration **(D)** of risk assessments as compared to benign odor. In contrast, female* Foxp2*^+/–^ mice do not display risk assessment behavior **(C,D)**. Ctr female mice also display stereotypical escape behaviors to rat bedding, observed as a significant increase in the number of climbs **(E)** and duration of climbing **(F)** compared to benign odor. In contrast, this escape behavior is not observed in female *Foxp2*^+/–^ mice **(E,F)**. The experiment was repeated in four different cohorts (total: *Foxp2*^+/–^ *n* = 10, ctr *n* = 10). p* ≤ 0.05; p** ≤ 0.01. NS, not significant.

Predator odor avoidance is an innate behavior in which an animal displays avoidance to olfactory cues of a predator, and when in a confined environment this behavior is characterized by increased risk assessment (cautious investigation) and decreased sniffing of predator olfactory cue followed by stereotypical escape responses such as climbing (Apfelbach et al., 2005). In this assay, we used rat bedding as a predator odor, which is typically highly aversive to mice (Sokolowski et al., 2015; Figure 7). In our hands, control male mice did not display the typical avoidance response (data not shown), whereas female control mice displayed the stereotypical reduced sniffing and increased risk assessment behavior and climbs (Figure 7). Therefore, here we present only the data for females. *Foxp2*^+/–^ female mice displayed an overall diminished predator avoidance response as indicated by no change in risk assessment, sniffing, and/or climbs in the presence of rat odor compared to benign odor. This indicates that *Foxp2*^+/–^ mutant females did not interpret rat odor as aversive.

In sum, we found that *Foxp2*^+/–^ mice displayed impaired social interaction, olfactory discrimination/motivation, mating/reproduction, aggression against an intruder, and predator odor avoidance, with social interaction and aggression manifesting in a sex-specific manner (Table 1).

**Table 1:** Summary of behavioral changes in *Foxp2*^+/–^ mice.

Behavior	female	male
Social interaction	↓	NC
Olfactory Habituation/Dishabituation	⇊	↓
Mating	↓	⇊
Aggression	↑	↓
Parenting	↓	ND
Predator Odor Avoidance	↓	ND

*NC, no change; ND, not determined; Downward arrows reflect a significant decrease in the presentation of a given behavior, while upward arrows indicate an significant increase in the presentation of behavior. Double arrows indicate a stronger phenotype. A subset of behavioral changes differed in either the magnitude (olfactory habituation/dishabituation, mating) or presentation (aggression) between Foxp2^+/–^ female and male mice*.

### MeA Proteomic Changes in Female and Male *Foxp2*^+/–^ Mice

**Figure 8:**
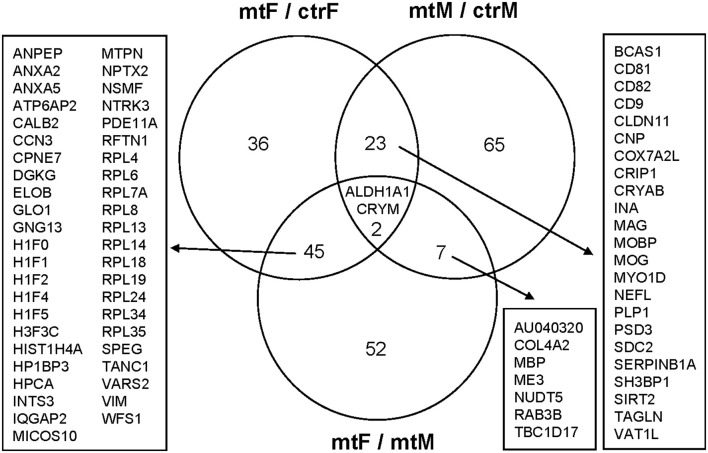
Overlapping and non-overlapping changes in protein expression in the MeA of female and male *Foxp2*^+/–^ mice. Venn Diagram of the differentially expressed proteins in the three comparisons from Figure 8: *Foxp2*^+/–^ female vs. ctr female (mtF/ctrF), *Foxp2*^+/–^ male vs. ctr male (mtM/ctrM), and *Foxp2*^+/–^ female vs. *Foxp2*^+/–^ male (mtF/mtM; Log2F C≥ |1.11|). The signature of proteins and neuromodulators in the amygdala of *Foxp2*^+/–^ mice was sex-specific.

**Figure 9:**
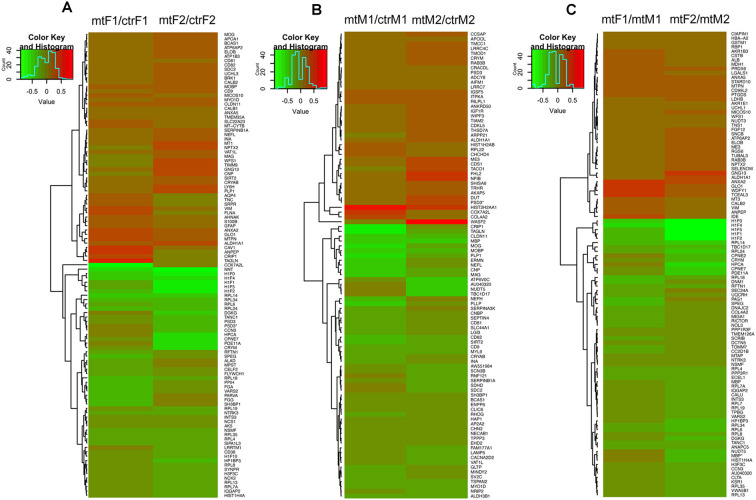
Heatmaps of differentially expressed proteins in the MeA of female and male *Foxp2*^+/–^ mice. Pooled microdissected MeA tissue from thee brains was run in duplicate. Ratios for each duplicate are shown in the x-axis and protein names (symbols) of differentially expressed proteins are displayed on the y-axis on the right. Panels **(A–C)** show proteins significantly upregulated (red) or downregulated (green) in three comparisons: *Foxp2*^+/–^ female vs. ctr female (mtF/ctrF) **(A)**, *Foxp2*^+/–^ male vs. ctr male (mtM/ctrM) **(B)**, *Foxp2*^+/–^ female vs. *Foxp2*^+/–^ male (mtF/mtM) **(C)** (Log2 fold change ≥ |1.11|). Proteins were clustered based on similarity of expression between the experimental and control groups, which is indicated by the dendrogram. The color key and histogram panel indicates the *Z*-scores of expression and the expression counts. See Supplementary Table 1 for a full list of altered proteins in each comparison.

As we observed robust differences in multiple behaviors in *Foxp2*^+/–^ mutant mice, with a subset displaying in a sex-specific manner, we next explored underlying proteomic changes correlating with these behavioral alterations in males and females. Based on the known role of the MeA in each of the social and non-social behaviors we tested (Kling and Brothers, 1992; Choleris et al., 2007; Sokolowski and Corbin, 2012; Bergan et al., 2014) and that the MeA is comprised of a large population of Foxp2+ cells (Carney et al., 2010; Lischinsky et al., 2017), we focused our proteomic analysis on this structure. MeA tissue from three animals (two amygdala/animal for a total of *n* = 6) was pooled together and considered a single sample. A total of *n* = 2 samples were analyzed for each sex (male and female) and genotype (*Foxp2*^+/–^ and control). We conducted TMT Isobaric Mass Tagging proteomics analysis to assess the full repertoire of proteins differentially expressed in the MeA of female and male *Foxp2*^+/–^ mutant mice. We compared female and male *Foxp2*^+/–^ mutants to their respective controls in three sets of analyses (Figures 8, 9). First, comparing female *Foxp2*^+/–^ mutants to female control, we observed 107 differentially expressed proteins (Figure 9A, Supplementary Table 1). Second, comparing male *Foxp2*^+/–^ mutants to male control, we observed 97 differentially expressed proteins (Figure 9B, Supplementary Table 1). Third, comparing male *Foxp2*^+/–^ mutants to female *Foxp2*^+/–^ mutants, we observed 107 differentially expressed proteins (Figure 9C, Supplementary Table 1). Two proteins, ALDH1A1 and CRYM, were found in all comparisons, with other protein sets common to two comparisons or only in one comparison (Figure 9). In addition to protein changes that are common across more than one comparison, a large number of protein changes were solely observed in each comparison (36 for *Foxp2*^+/–^ mutant F vs. control F; 64 for *Foxp2*^+/–^ mutant M vs. control M; 52 for *Foxp2*^+/–^ mutant M vs. *Foxp2*^+/–^ mutant F). Thus, these protein sets represent both mutant vs. control differences within the same sex, as well as sex-specific signatures of *Foxp2* function in the MeA. It is important to note that as we performed global quantitative proteomics analyses in only two technical replicates, these results will need future replication in larger sample sets and validation of candidates of interest on tissue. Despite this caveat, as described below, our analysis suggests alterations in interesting pathways.

**Figure 10:**
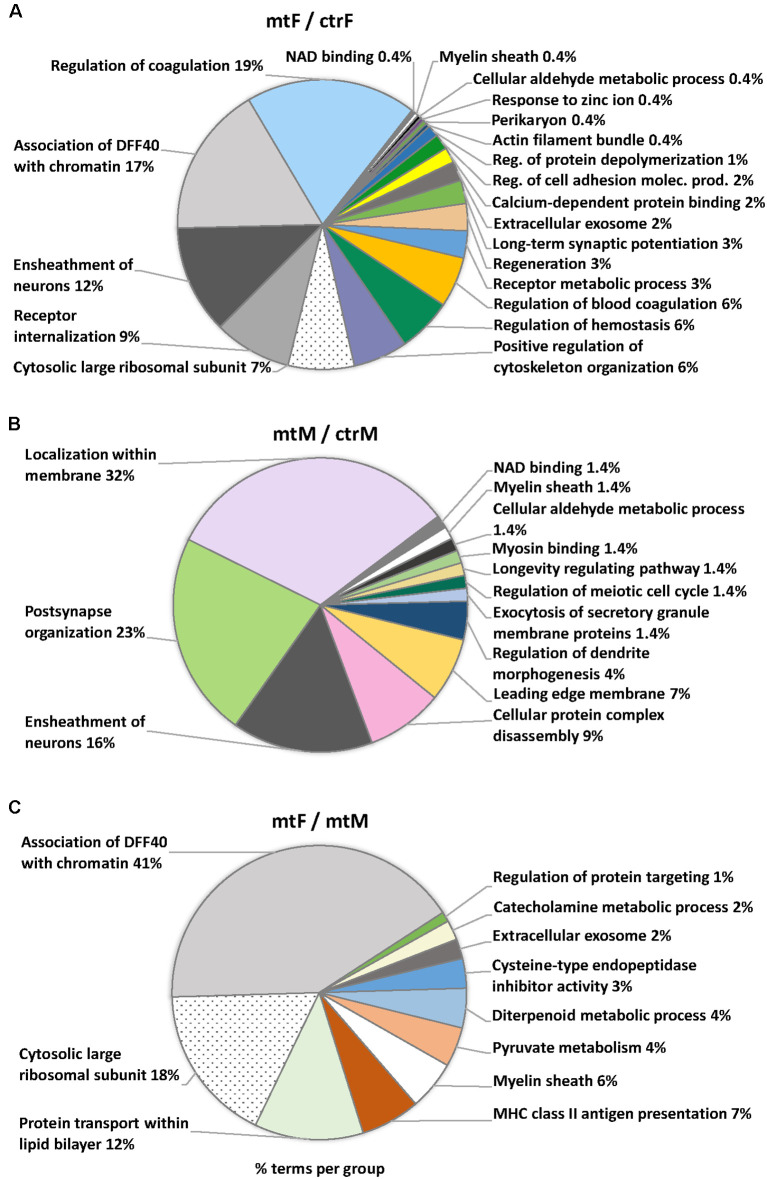
Pie charts of enriched Ontology (GO) terms for the proteins differentially expressed in the MeA of female and male *Foxp2*^+/–^ mice. Ontology analysis in the three comparisons: *Foxp2*^+/–^ female vs. ctr female (mtF/ctrF), *Foxp2*^+/–^ male vs. ctr male (mtM/ctrM), *Foxp2*^+/–^ female vs. *Foxp2*^+/–^ male (mtF/mtM). Myelin sheath was a common term for the three comparisons (colored in white). Common terms across (**A,B**; i.e., Ensheathment of neurons, NAD binding, Cellular aldehyde metabolic process) and (**A,C**; Association of DFF40 with chromatin, Cytosolic large ribosomal subunit, Extracellular exosome) are colored in scales of gray. Selected terms and related metrics were determined using ClueGO, based on the terms per group with the highest percentages.

Enriched ontology (GO) analyses revealed common pathway alterations in neuronal communication and protein transport (Figure 10, Table 2). Indeed, within the enriched GO terms for the proteins differentially expressed in the MeA across comparisons, the term “myelin sheath” was common across all three comparisons, and six other terms were common in two comparisons: “NAD binding”, “ensheathment of neurons” and “cellular aldehyde metabolic process” (in comparisons Figures 10A,B), and “association of DFF40 with chromatin”, “cytosolic large ribosomal subunit” and “extracellular exosome” (in comparisons Figures 10A,C). Among them, “association of DFF40 with chromatin” was the most enriched term (41%), when comparing female to male mutants (Figure 10). Other prominent GO-enriched terms based on the terms per group with the highest percentages were related to chromatin regulation, cell cycle, and catecholamine metabolic process (Table 2).

**Table 2:** Enriched ontology GO terms and associated proteins.

	GO Term	List of associated proteins
mtF/CtrF	Regulation of coagulation	[ANXA2, ANXA5, CAV1, CD9, FGA, FGG]
	Association of DFF40 with chromatin	[H1F0, HIST1H1A, HIST1H1B, HIST1H1C, HIST1H1E]
	Ensheathment of neurons	[BCAS1, CD9, CLDN11, MAG, NTRK3, PLP1, S100B, SIRT2]
	Receptor internalization	[CAV1, CD81, CD9, HPCA, LRRTM1, SIRT2]
	Cytosolic large ribosomal subunit	[RPL13, RPL14, RPL18, RPL19, RPL24, RPL34, RPL35, RPL4, RPL6, RPL7A, RPL8]
	Regulation of hemostasis	[ANXA2, ANXA5, CAV1, CD9, FGA, FGG]
	Positive regulation of cytoskeleton organization	[APOA1, BRK1, CAV1, FLNA, IQGAP2, NCK2, NTRK3, PLP1]
	Regeneration	[APOA1, CCN3, CD81, CD9, GFAP, MAG, MTPN, NEFL, NTRK3, TNC]
	Receptor metabolic process	[ANXA2, CAV1, CD81, CD9, HPCA, LRRTM1, SIRT2]
	Long-term synaptic potentiation	[CALB1, CALB2, GFAP, LRRTM1, S100B]
	Extracellular exosome	[AHNAK, ANPEP, ANXA2, CD81, CD9]
	Regulation of cell adhesion molecule production	[APOA1, AQP4, CAV1]
	Calcium-dependent protein binding	[ANXA2, MYO1D, NSMF, S100B, WFS1]
	Regulation of protein depolymerization	[MTPN, PLP1, SH3BP1]
	Response to zinc ion	[ALAD, CRIP1, MT1]
	Myelin sheath	[ANXA2, CLDN11, CNP, CRYAB, GFAP, INA, MAG, MOBP, MOG, MYO1D, NEFL, PLP1, SIRT2]
	NAD binding	[ALDH1A1, NNT, SIRT2]
	Cellular aldehyde metabolic process	[ALDH1A1, GLO1, PLP1]
	Perikaryon	[ANXA5, CRYAB, HPCA, MYO1D, NSMF, PDE11A, SIRT2]
	Actin filament bundle	[CRYAB, FLNA, SIPA1L3]
mtM/CtrM	Localization within membrane	[AKAP5, CD81, HAP1, LRRC7, RILPL1, SHISA6, SIRT2]
	Postsynapse organization	[CDKL5, IGF1R, INA, ITPKA, NEFH, NEFL, SHISA6, WASF2]
	Ensheathment of neurons	[BCAS1, CD9, CLDN11, MAG, MBP, PLLP, PLP1, SIRT2, TSPAN2]
	Cellular protein complex disassembly	[AKAP5, CCSAP, PLP1, SH3BP1, TMOD1]
	Leading edge membrane	[AKAP5, CDKL5, LAMP5, MYO1D, PLP1, PSD3, SHISA6]
	Regulation of dendrite morphogenesis	[AKAP5, CDKL5, ITPKA, SDC2]
	Myelin sheath	[CLDN11, CNP, CRYAB, EHD2, ERMN, INA, MAG, MBP, MOBP, MOG, MYO1D, NEFH, NEFL, PLLP, PLP1, SIRT2, TSPAN2]
	Myosin binding	[HAP1, MOBP, MYL9, RAB3B]
	Longevity regulating pathway	[ADCY8, CRYAB, IGF1R]
	Regulation of meiotic cell cycle	[CNP, IGF1R, SIRT2]
	NAD binding	[ALDH1A1, ME3, SIRT2]
	Cellular aldehyde metabolic process	[ALDH1A1, ALDH3B1, PLP1]
	Exocytosis of secretory granule membrane proteins	[ALDH3B1, AP2A2, RHOG]
mtF/mtM	Association of DFF40 with chromatin	[H1F0, HIST1H1A, HIST1H1B, HIST1H1C, HIST1H1E]
	Cytosolic large ribosomal subunit	[RPL13, RPL14, RPL18, RPL19, RPL24, RPL34, RPL35, RPL4, RPL6, RPL7, RPL7A, RPL8]
	Protein transport within lipid bilayer	[DNM1, HPCA, PPP3R1, RFTN1, SCRIB]
	MHC class II antigen presentation	[CLTA, DCTN5, DNM1, SEC24A, TUBAL3]
	Myelin sheath	[AKR1B3, ALB, ANXA2, DNM1, GSTM1, LDHB, MBP, MDH1, SCRIB, UCHL1]
	Diterpenoid metabolic process	[AKR1B3, ALDH1A1, RBP1]
	Pyruvate metabolism	[GLO1, LDHB, MDH1, ME3]
	Cysteine-type endopeptidase inhibitor activity	[CSTB, MT3, NOL3, PRDX6]
	Catecholamine metabolic process	[AKR1B3, MTPN, SNCB]
	Extracellular exosome	[ALB, ANPEP, ANXA2, IDE]
	Regulation of protein targeting	[HPCA, NOL3, TOMM7]

*Listed proteins differentially expressed in the MeA of Foxp2+/− mice associated to the enriched ontology terms (in Figure 10) with the highest percentages using ClueGO*.

One interpretation of our above described behavioral analyses (Figures 1–7) is that deficits in *Foxp2*^+/–^ mutant behavior may reflect an alteration in the motivational drive. As dopamine (DA) is a prime neurotransmitter implicated in motivational states and previous studies have revealed dopaminergic inputs to the MeA (Poulin et al., 2018), we assessed whether there were protein changes in the expression of known DA pathway/DA regulated proteins (Table 3). Across comparisons, we found 3 DA pathway proteins to have altered expression (FLNA, GFAP, DMN1).

**Table 3:** Detailed information on the biological function of the 15 ASD and three dopamine related proteins differentially expressed in the MeA.

Symbol	Comp.	Related	Cat.	Description	References
BCAS1	1,2	ASD	3	**Brain Enriched Myelin Associated Protein 1** associates with a variety of aggressive tumors. In the brain, BCAS1 isrequired for myelination and is expressed in oligodendrocytes and Schwann cells, showing decreased expression in demyelination. Mice lacking BCAS1 showed schizophrenia-like behaviors. Exome sequencing confirmed association with autism.	Ishimoto et al. (2017)
CD38	1	ASD	3	**CD38** is an ectoenzyme widely expressed (especially in leukocytes), with functions in calcium transportation and signaling, cell adhesion, signal transduction and regulation of oxytocin and paternal behavior. CD38 expression was significantly reduced in ASD subjects. Genetic association to ASD was found in AGRE, Japanese and Israeli cohorts.	Martucci et al. (2019)
CD99L2	3	ASD	3	**CD99 Molecule Like 2** is a cell-surface protein similar to CD99 and plays a role in leukocyte extravasation, helping immune cell transmigration through the endothelial basement membrane. As in other immune-related genes, SNPs in CD99L2 were significantly associated with ASD.	Ramos et al. (2012)
CDKL5	2	ASD	1	**Cyclin Dependent Kinase Like 5** is a member of Ser/Thr protein kinase family involved in the phosporilation of proteins, especially important in the posttranslational modifications. In mice, CDKL5 deficiency compromised the GABA/Glut balance.Mutations in CDKL5 associates with syndromic autism, Rett syndrome, Angelman and seizures.	Tang et al. (2019)
ERMN	2	ASD	3	**Ermin** is an oligodendrocyte cytoskeletal protein involved in myelination. Deficient myelination has been reported in ASD subjects. Hypomethylation caused by genetic variants at ERMN significantly associated with ASD.	Galvez-Contreras et al. (2020)
FGA	1	ASD	3	**Fibrinogen Alpha Chain**, the alpha subunit of the coagulation factor fibrinogen, is a component of the blood clot also involved in the stabilization of the lesion and cell migration guidance. Several SNPs in FGA showed association with ASD.	Yang et al. (2018)
GLO1	1,3	ASD	3	**Glyoxalase I** mediates catalysis and formation of S-lactoyl-glutathione from methylglyoxal (MG) and reduced glutathione, decreasing MG levels. MG is a precursor of advanced glycation end products (AGE) and both MG and AGEs can induce oxidative stress, mitochondrial dysfunction and inflammation. High AGE levels were found in the brain of ASD subjects.	Kovač et al. (2015)
GSTM1	3	ASD	3	**Glutathione S-Transferase Mu 1** functions in the detoxification of electrophilic compounds including drugs and environmental toxins, by conjugating with glutathione, an antioxidant acting as a free radical scavenger. Deficits in GSTM1 may increase sensitivity to toxicant exposure early in life, which has been pointed out to trigger ASD.	Yochum et al. (2010)
LRRC4C	2	ASD	1-Satt.	**Leucine Rich Repeat Containing 4C** is a specific binding partner for netrin G1 (NTNG1), a member of the netrin family of axon guidance molecules. LRRC4C plays a central role in early nervous system development and differentiation, and it may promote neurite outgrowth. Genetic variants in LRRC4C have been detected in ASD subjects.	Um et al. (2018)
NFIB	2	ASD	Synd.	**Nuclear Factor I B** is a transcription factor essential in embryonic development. NFIB acts as a cofactor of FOXP2 to activate genes involved in neuronal maturation and is a transcriptional activator of GFAP, essential for brain development. Mutations in NFIB led to intellectual disability, speech delay, macrocephaly, behavioral deficits and ASD.	Hickey et al. (2019)
NTRK3	1,3	ASD	3	**Neurotrophic Receptor Tyrosine Kinase 3** is a membrane-bound receptor that, upon neurotrophin binding, phosphorylates itself and members of the MAPK pathway that control cell survival and differentiation. Genetic association of NTRK3 has been found with autism and Asperger syndrome.	Chakrabarti et al. (2009)
PSD3	1,2	ASD	3	**Pleckstrin And Sec7 Domain Containing 3** is a guanine nucleotide exchange factor for ARF6 and is involved in phospholipid binding and endocytosis. Rare mutations involving PSD3 have been identified in individuals with ASD.	Pinto et al. (2010)
SDC2	1,2	ASD	3	**Syndecan 2** is a transmembrane heparan sulfate proteoglycan. The syndecans mediate cell binding, cell signaling, cytoskeletal organization, cell proliferation and cell migration. In neurons, SDC2 is highly expressed during development and is involved in spine and synapse formation. A rare mutation in the SDC2 gene has been identified in autism.	Hu et al. (2016)
SRPR	1	ASD	Satt.	**SRP Receptor Subunit Alpha** is a component of the SRP (signal recognition particle) receptor of the Endoplasmic Reticulum that ensures the correct targeting of the nascent secretory proteins to the endoplasmic reticulum membrane system. In autism, its expression in the cortex may be regulated by ASD-associated SNPs through DNA methylation.	Sun et al. (2019)
GFAP	1	ASD /DOPA	1-Satt.	**Fibrous astrocytes Glial Fibrillary Acidic Protein** is a major intermediate filament protein of mature astrocytes, common marker of astrocytes and a target of dopamine. It is involved in responses to brain inflammation, injury and disease. Astrocytic abnormalities and increased mRNA levels of *GFAP* have been observed in post-mortem brains of autistic individuals.	Edmonson et al. (2014)
DNM1	3	DOPA		**Dynamin 1** is a GTP-binding protein important for synaptic vesicle endocytosis in neurotransmission that exhibits D2 dopamine receptor binding. Genetic variants in DNM1 are one of the most common causes of epileptic encephalopathy.	Bonnycastle (2018)
FLNA	1	DOPA		**Filamin A** promotes branching of actin filaments and anchors transmembrane proteins to the cytoskeleton. In the brain, FLNA plays a role in cell-cell contacts and adherens junctions during the development and intervenes in the internalization of transmembrane receptors such as dopamine receptor type 2 and 3 (DRD2 and DRD3). FLNA is also involved in neuronal migration and it is required for growth cone collapse in axons.	Coelho et al. (2019)

*Column “Comp.” states in which comparison(s) each of these proteins were differentially expressed (i.e., 1: *Foxp2*^+/–^ female vs. ctr female; 2: Foxp2+/− male vs. ctr male; 3: *Foxp2*^+/–^ female vs. *Foxp2*^+/–^ male). ASD-related proteins were classified according to SFARI categories (syndromic; 1: high confidence; 2: strong candidate, and 3: suggestive evidence) and/or present in the largest exome sequencing study of ASD individuals by Satterstrom et al. (2020). From the list, GFAP was the only protein related to both ASD (Cat. 1/Satt.) and dopamine signaling; and NF1B was the only one classified as “syndromic”. Information in the table was summarized from Genecards and SFARI websites, as well as additional references. Expression of CD38, FGA, NTRK3, and GFAP had been previously reported to be sexually dimorphic in the brain (Jin et al., 2007; Berchtold et al., 2008; Arias et al., 2009; Ramsey et al., 2012); and CDKL5 disorders to primarily affect females (Bahi-Buisson and Bienvenu, 2011)*.

Mutations in *FOXP2* in humans have been consistently associated with Autism Spectrum Disorders (ASD), and as such, *FOXP2* is ranked category 1 (high confidence) in the SFARI autism gene database^1^. Comparing proteomic changes in *Foxp2*^+/–^ mutant mice to both the SFARI gene database and the results of the most comprehensive ASD sequencing effort to date (Satterstrom et al., 2020), we found 15 proteins implicated in ASD (Table 3) in at least one comparison, with one of these, the nuclear factor NFIB, being syndromic.

### Sex Differences in Dopamine Activation of *Foxp2*-Lineage Cells in the MeA

**Figure 11:**
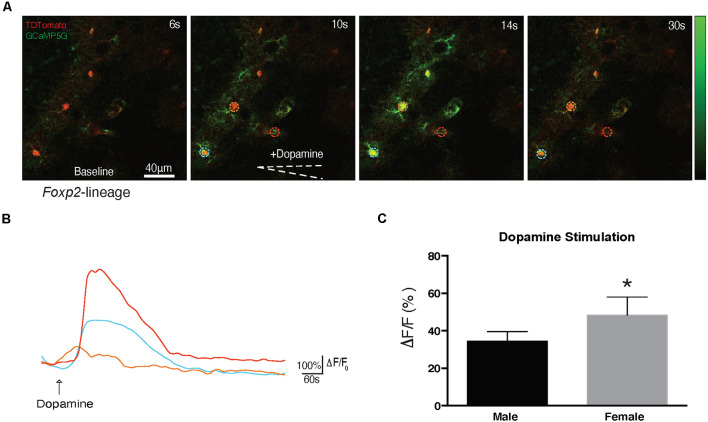
Foxp2-lineage cells in MeA are more responsive to dopamine in females. Dopamine evoked Ca^2+^ transients were assessed in 300 μm MeA slices from *Foxp2^cre^*;*GCaMP5G-tdTM* mice. Time series of Ca^2+^ increases in *Foxp2*-lineage cells by focal application of dopamine (100 mM) from a glass pipette (white dotted line) is shown: in red the recombined Foxp2-lineage cells, in green the Ca++ transient **(A)**. The pseudocolor scale displays changes in GCaMP5G △F/F. Representative individual traces of GCaMP5G fluorescence changes (△F/F) in response to dopamine **(B)**. The color-coding of the traces matches the ROI of the colored circles in **(A)**. Dopamine evoked significantly more activation in *Foxp2*-lineage cells in females than in males **(C)**. Analysis was carried out in *n* = 5 females, *n* = 4 males in at least two slices per animal containing the MeA. *p ≤ 0.05 (*p* = 0.0294).

Previous studies have identified DA-ergic inputs to the MeA (Poulin et al., 2018). The above described proteomic analysis revealed putative alterations in DA signaling, however, it is not known if Foxp2+ cells can respond directly to DA. To test this, we crossed *Foxp2^cre^* mice (Rousso et al., 2016) to *GCaMP5G-tdTM* reporter mice (Gee et al., 2014) allowing us to assess evoked Ca^2+^ transients in Foxp2+ cells in acute brain slices (Figures 11A,B). Focal application of DA (100 μm) evoked cytosolic Ca^2+^ increases in multiple morphologically distinct cell types resembling astrocytes, neurons, and ependyma. We further found Foxp2+ activation patterns in response to DA differed in females and males, with less activation in females (Figure 11C). Thus, this analysis reveals that MeA Foxp2+ cells can be activated by dopamine, a neurotransmitter involved in social behavior, motivation, and reward.

## Discussion

In this study, we found that heterozygous* Foxp2*^+/–^ knockout mice display altered behavior across a host of innate social and non-social behaviors (summarized in Table 1). The brain regions modulating these behaviors are well documented and most prominently include the olfactory bulb (both main and accessory), the cortical and medial nuclei of the amygdala (MeA), bed nucleus of the stria terminalis (BNST), and multiple nuclei of the hypothalamus (Gross and Canteras, 2012; Sokolowski and Corbin, 2012; Chen and Hong, 2018; Li and Dulac, 2018). Of these regions, Foxp2+ cells are found in high densities in the olfactory bulb (both main and accessory), and MeA, with fewer cells in the hypothalamus^2^. Thus, Foxp2+ neurons are well-positioned to modulate social and non-social innate behaviors, and deficits in *Foxp2* gene function in any of these regions may underlie the innate behavioral deficits observed here. Our previous work (Lischinsky et al., 2017) revealed that the Foxp2+ cells of the MeA comprise a subpopulation of inhibitory output neurons. As the MeA is directly involved in processing olfactory sensory information for appropriate behavioral outputs, largely *via* connections with the BNST and hypothalamus, the MeA represents a likely site of deficit for the altered behavioral processing we observed in *Foxp2*^+/–^ mice. Consistent with this role, *via* cFos expression, we previously observed that MeA Foxp2+ cells respond to a host of innate behavioral cues (Lischinsky et al., 2017). However, it is important to note that while we hypothesize that deficits in the MeA are at least in part causally linked to our behavioral phenotypes observed in *Foxp2*^+/–^ mice, deficits in other brain regions cannot be ruled out at this time. Conditional *Foxp2* mutagenesis in the olfactory bulb, MeA, and hypothalamus will likely prove highly informative in dissecting the region-specific role of *Foxp2* in innate behavioral processing.

Of the behaviors we explored, we interestingly found sex differences mainly in two: social interaction and aggression. We observed decreased social interaction in female *Foxp2*^+/–^ mice, with no change in male *Foxp2*^+/–^ mice. Furthermore, and perhaps most striking, were the opposite consequences on aggressive behavior. Female *Foxp2*^+/–^ mice displayed enhanced aggression, while male *Foxp2*^+/–^ mice displayed decreased aggression. Although it is important to note that male territorial aggression is considered offensive aggression, while maternal aggression is considered defensive, we find the opposite results in *Foxp2*^+/–^ mice particularly intriguing. Thus, it appears that *Foxp2* gene function plays behaviorally separable roles in the male and female brain. As aggression manifests differently and in different contexts in male and female mice, this result may reflect either male/female differences in Foxp2+ circuitry and/or neuronal function. Consistent with this, the core aggression circuit across species is sexually dimorphic (Hashikawa et al., 2018; Li and Dulac, 2018). In mice, there are known sex differences in olfactory inputs to the MeA, as well as sex differences in MeA neuronal population activation in response to various olfactory cues. In the fruit fly, *Drosophila*, recent studies have revealed that males and females have different neurons for regulating aggression (Chiu et al., 2021). Although the circuit and/or neuronal mechanism of sex differences in aggressive and social interactive behaviors that we observed in *Foxp2*^+/–^ mice remains to be uncovered, our proteomic analyses provide us preliminary clues. The major difference when comparing female to male mutants is in the GO term “association with DFF40 with chromatin.” DFF40 is associated with apoptotic cell death (Liu et al., 1998), perhaps indicating male/female differences in the requirement for *Foxp2* in cell survival/apoptosis either in a cell-autonomous or non-cell-autonomous manner. In addition, based on our GO analyses, several other *Foxp2*-dependent processes may be involved, such as membrane protein transport and cytosolic large ribosomal transport, which together may suggest sex differences in protein trafficking and/or translation. In agreement with our results, extensive previous work has implicated *Foxp2* gene function in the multi-fold processes of chromatin regulation, activity-dependent plasticity, and dendritic morphology (French and Fisher, 2014; Co et al., 2020). As the MeA is a highly sexually dimorphic brain region (Gegenhuber and Tollkuhn, 2020; Matos et al., 2020), it is possible that *Foxp2* may be required to regulate these processes differently in males and females. Unraveling the underlying neuronal and circuit mechanisms of the *Foxp2*-dependent control of innate behaviors as well as the sex differences in the function of *Foxp2* will likely be an exciting area of future investigation.

Our extensive behavioral analyses of *Foxp2*^+/–^ mice reveal robust deficits across a number of innate behavioral domains, typically displayed as a blunted behavioral response across various behaviors (with the notable exception of aggression in female *Foxp2*^+/–^ mice, which is enhanced). One interpretation of this data is that *Foxp2* is required for proper motivation to execute a given behavior. In this context, one apparent candidate pathway to explain our behavioral data would be alterations in dopamine (DA) signaling, which is highly implicated in motivation and reward-seeking behavior. Focusing on DA signaling, from our proteomics analyses, we found that *Foxp2*^+/–^ mice have alterations in the expression of DA-signalling/DA-responsive proteins FLNA, GFAP, and DMN1. However, as our proteomics analysis was done on whole MeA tissue, thus containing both Foxp2-positive and Foxp2-negative cells, we cannot determine if this deficit is cell-autonomous or cell non-autonomous. To investigate whether Foxp2+ cells can directly respond to DA, we show *via* 2-Photon Ca^2+^ imaging that Foxp2+ cells are highly responsive to DA, and notably with differences between males and females. It will be interesting to explore if/how DA signaling is altered in Foxp2+ cells in *Foxp2*^+/–^ mutants. In the striatum, Foxp2 is expressed in many medium spiny neurons, major targets of dopaminergic inputs (Ferland et al., 2003; Lai et al., 2003; Takahashi et al., 2003; Scharff and Haesler, 2005; Wijchers et al., 2006). Consistent with a putative *Foxp2*-dependent role for DA, Enard et al. (2009) observed increased DA levels in heterozygous *Foxp2*^+/–^ mice across a number of brain regions (though this study did not include the amygdala). In addition, while the cellular target was not identified, previous axonal tracing studies have shown that there are major DA inputs to the MeA (Poulin et al., 2018), and MeA neurons express DR1 receptors (Miller et al., 2019). Consistent with this, in humans, dopamine modulates the amygdala network (also comprising some areas of the prefrontal cortex and the nucleus accumbens) to mediate mother-infant bonding (Atzil et al., 2017). It is therefore plausible that alterations in DA signaling may be a contributing component to the behavioral deficits we observed in *Foxp2*^+/–^ mice.

In humans, *FOXP2* has been most closely linked to language disorders (Morgan et al., 2017). Despite conflicting genetic evidence, many studies have found an association between *FOXP2* mutations and ASD (Gong et al., 2004; Li et al., 2005; Marui et al., 2005; Feuk et al., 2006; Laroche et al., 2008; Chien et al., 2017; Lim et al., 2017; Guo et al., 2019; Munnich et al., 2019; Satterstrom et al., 2020). ASD is a common multi-genic neurodevelopmental disorder affecting 1 in 68 individuals (with a higher prevalence in males), with little known regarding the underlying neurobiology of sex differences in diagnosis. We interestingly observed that *Foxp2*^+/–^ female, but not male, mice display deficits in the 3-chambered social task. This task is one of the major assays for face validity in ASD-animal models, suggesting ASD-like behavior in female *Foxp2*^+/–^ mice. Consistent with this, we found that in the MeA, *Foxp2*^+/–^ mice differentially expressed 15 ASD-risk proteins. This is perhaps not surprising as amygdala dysfunction has been highly implicated in the pathophysiology of ASD (Baron-Cohen et al., 2000; Lim et al., 2017; Avino et al., 2018). One protein of particular interest is the syndromic nuclear factor NFIB, which was upregulated in *Foxp2*^+/–^ males. This protein is a coactivator of FOXP2 and is able to regulate androgen and estrogen receptor activity (Grabowska et al., 2014; Becker-Santos et al., 2017; Hickey et al., 2019). Another interesting protein is GFAP, a common marker for astrocytes which is also related to both ASD (SFARI: Score 1 = high-confidence; Edmonson et al., 2014; Herrero et al., 2020; Satterstrom et al., 2020) and dopamine signaling (Sands and Chronwall, 1996; Galloway et al., 2018). Notably, GFAP was only differentially expressed when comparing *Foxp2*^+/–^ mutant females vs. ctr females, perhaps suggesting a role for this protein in the sex-specific differences associated with deficiency of *Foxp2*^+/–^ in females. Thus, while *Foxp2*^+/–^ mice are not a model for syndromic ASD, our findings may provide a window into differential gene and protein regulation in the male and female brain in relation to ASD.

## Data Availability Statement

The original contributions presented in the study are included in the article/Supplementary Material, further inquiries can be directed to the corresponding author/s. The mass spectrometry proteomics data have been deposited to the ProteomeXchange Consortium *via* the PRIDE partner repository (Perez-Riverol et al., 2019) with the dataset identifier PXD026978.

## Ethics Statement

The animal study was reviewed and approved by Children’s National Hospital Institutional Animal Care and Use Committee (IACUC), conformed to NIH Guidelines for animal use.

## Author Contributions

MH: conceptualized the study, conducted microdissections, protein isolation, oversaw proteomic screen and with AP analyzed the proteomics data; managed the mouse colony and coordinated behavioral experiments with LW, and co-wrote the manuscript with JC. LW: designed and conducted the experiments on behavior, data analysis, and co-wrote behavior materials and methods and results. DH-P: animal care and handling and assisted with data analysis. PB: assisted with proteomic data analysis and plotting heatmaps. HM: assisted in calcium imaging experiment and brain slice preparation. MG: assisted with animal colony maintenance and genotyping. AP: oversaw proteomic quantitation, conducted mass spec experiments and with MH proteomic data analysis. NS: conducted experiment on calcium imaging, data analysis, and writing of materials and methods and results. JC: conceptualized and supervised the study, managed collaborations, provided funding, and co-wrote the manuscript with MH. All authors contributed to the article and approved the submitted version.

## Conflict of Interest

The authors declare that the research was conducted in the absence of any commercial or financial relationships that could be construed as a potential conflict of interest.

## Publisher’s Note

All claims expressed in this article are solely those of the authors and do not necessarily represent those of their affiliated organizations, or those of the publisher, the editors and the reviewers. Any product that may be evaluated in this article, or claim that may be made by its manufacturer, is not guaranteed or endorsed by the publisher.

## Footnotes

^1^ https://gene.sfari.org/database/human-gene/FOXP2

^2^ http://mouse.brain-map.org/gene/show/76994

## Supplementary Material

Supplementary Table 1 List of differentially expressed proteins in the MeA of *Foxp2*^+/–^ female and male mice. Differentially expressed proteins are listed from the three comparisons: *Foxp2*^+/–^ female vs. ctr female (mtF/ctrF), *Foxp2*^+/–^ male vs. ctr male (mtM/ctrM), *Foxp2*^+/–^ female vs. *Foxp2*^+/–^ male (mtF/mtM). The total number of differentially expressed proteins for each of the three comparisons was respectively: 107 proteins (54 downregulated, 53 upregulated); 97 proteins (57 downregulated, 40 upregulated), and 107 proteins (64 downregulated, 43 upregulated).
